# Clustering Methods for Vibro-Acoustic Sensing Features as a Potential Approach to Tissue Characterisation in Robot-Assisted Interventions

**DOI:** 10.3390/s23239297

**Published:** 2023-11-21

**Authors:** Robin Urrutia, Diego Espejo, Natalia Evens, Montserrat Guerra, Thomas Sühn, Axel Boese, Christian Hansen, Patricio Fuentealba, Alfredo Illanes, Victor Poblete

**Affiliations:** 1 Instituto de Acústica, Facultad de Ciencias de la Ingeniería, Universidad Austral de Chile, Valdivia 5111187, Chile; robin.urrutia@alumnos.uach.cl (R.U.); vpoblete@uach.cl (V.P.); 2 Audio Mining Laboratory (AuMiLab), Instituto de Acústica, Universidad Austral de Chile, Valdivia 5111187, Chile; diego.espejo@aumilab.cl; 3 Instituto de Anatomia, Histologia y Patologia, Facultad de Medicina, Universidad Austral de Chile, Valdivia 5111187, Chile; natalia.evens@alumnos.uach.cl (N.E.); monserratguerra@uach.cl (M.G.); 4 Department of Orthopaedic Surgery, Otto-von-Guericke University Magdeburg, 39120 Magdeburg, Germany; thomas.suehn@icloud.com; 5 SURAG Medical GmbH, 39118 Magdeburg, Germany; alfredo@surag-medical.com; 6 INKA Innovation Laboratory for Image Guided Therapy, Otto-von-Guericke University Magdeburg, 39120 Magdeburg, Germany; 7 Research Campus STIMULATE, Otto-von-Guericke University Magdeburg, 39106 Magdeburg, Germany; christian.hansen@ovgu.de; 8 Instituto de Electricidad y Electrónica, Facultad de Ciencias de la Ingeniería, Universidad Austral de Chile, Valdivia 5111187, Chile; pfuentealba@uach.cl

**Keywords:** robot-assisted surgery, dimensionality reduction, minimally invasive surgery, haptic information, vibration sensing, surgical data science, surgery augmentation, tissue classification

## Abstract

This article provides a comprehensive analysis of the feature extraction methods applied to vibro-acoustic signals (VA signals) in the context of robot-assisted interventions. The primary objective is to extract valuable information from these signals to understand tissue behaviour better and build upon prior research. This study is divided into three key stages: feature extraction using the Cepstrum Transform (CT), Mel-Frequency Cepstral Coefficients (MFCCs), and Fast Chirplet Transform (FCT); dimensionality reduction employing techniques such as Principal Component Analysis (PCA), t-Distributed Stochastic Neighbour Embedding (t-SNE), and Uniform Manifold Approximation and Projection (UMAP); and, finally, classification using a nearest neighbours classifier. The results demonstrate that using feature extraction techniques, especially the combination of CT and MFCC with dimensionality reduction algorithms, yields highly efficient outcomes. The classification metrics (Accuracy, Recall, and F1-score) approach 99%, and the clustering metric is 0.61. The performance of the CT–UMAP combination stands out in the evaluation metrics.

## 1. Introduction

Surgery is one of the medical fields that has witnessed significant technological advances in recent decades. Traditional surgery, although effective, can be a painful and invasive process for the patient and entails long recovery periods and potential complications. The introduction and increased use of Minimally Invasive Robotic Surgery (MIRS) in diverse medical operations have revolutionised surgical practice because of its benefits for patients, such as less postoperative pain and less invasive procedures [1,2,3]. Surgical robots have been designed to perform procedures using smaller incisions, resulting in reduced pain, inflammation, and potential complications by providing robustness to time-varying nonlinear incision trajectories and greater precision [4,5]. This technology, in turn, contributes to accelerating the patient’s recovery process [6,7]. Additionally, MIRS provides the surgeon with more precise visualisation and control, which can lead to a significant improvement in surgical outcomes [8,9].

Despite the benefits of MIRS, one challenge is the loss of a surgeon’s tactile sense [10,11]. Various techniques have been investigated to address this challenge, such as the use of force and contact sensors and the implementation of microsensors in surgical instruments to provide real-time haptic feedback [12,13,14].

These sensor techniques encounter significant challenges, mostly related to technical and regulatory requirements, such as those mentioned in [15,16,17,18], including (1) size, (2) cost, (3) integrability in terms of electrically active components, (4) sterilisability, (5) bio-compatibility, etc. In most cases, they stay in direct contact with the tissue surface inside the patient’s body [19,20]. For this reason, there is a need to discover new designs and manufacturing methods in order to provide high-quality patient surgical registries that are more accurate, consistent, and reproducible, and, therefore, to gain insight into the physical interaction between the target tissue surface and instrument tip.

In the last decade, the possibility of combining and fusing medical information of multi-modal data has shown great potential for the fast and accurate prediction of disease diagnostics. The reason for fusing information from multiple modalities is to provide complementary medical information from different sources about the same medical event (e.g., videos, images, sensor data, and algorithms or models) and to predict diseases with higher confidence, which otherwise could not have been detected individually (see, e.g., [21,22]).

One example of data fusion is the multi-modal imaging technique for brain pathology diagnosis, where the utilisation of computer vision algorithms as diagnostic tools for processing brain scan images has a direct impact on the patient’s life [23]. Feature information in medical images is usually unstructured and presents an important amount of noise. The extraction of the relevant features of brain tissues (e.g., image intensity, smooth shape, localisation, and inflammation with undefined edges), which helps to differentiate healthy tissue from pathological cases and perform subsequent diagnostics, requires several hours of work, even for a single case, by a highly specialised surgeon [24]. To overcome this drawback, machine learning with deep neural networks has shown great promise in the automatic classification of medical images, facilitating surgeon decisions and providing objectivity in these decisions [25].

In the traditional context of medical pattern recognition applied to automatic classification, which helps in clinical diagnosis and robot-assisted surgery, the general framework has at least two major components: feature engineering and feature-based classification algorithms [26]. The term feature engineering refers to an approach to creating numeric features of raw medical data to derive more informative measures [27]. In practice, this is not an easy task. There are some problems in finding an effective approach. On the one hand, manual feature extraction relies on surgeon experience and knowledge. On the other hand, manual features may not be adequate for specific medical operations compared to features directly learned from interventional and surgical data. Additionally, manual feature engineering is time consuming and tedious. The number of features is also important. If there is enough medical information, then feature-based classification algorithms will be able to perform the ultimate task. Furthermore, if most of the extracted features are irrelevant or are not good features, then the classification algorithm will be unable to perform the desired task. Good features make the classification component easy, and the resulting algorithm is more capable of supporting the surgeon’s task. To address the problem of manual feature extraction, the automated feature extraction of medical data reduces the dependency on manual feature engineering and the prior knowledge of analysis of the surgeon. An ideal automated feature extractor offers the allure of being able to extract relations between data with little effort by the surgeon. The utilisation of exploratory visualisations of the medical data helps guide feature engineering. Spending time exploring the complex structures between features leads to better understandings of the medical data. Therefore, data visualisations are essential tools of feature engineering [28].

Two other examples in the literature, about fusing surgical information from multiple modalities, are based on the use of sound and vibration waves. Firstly, Wang et al. [29] suggested that during robot-assisted cervical surgery, the high-speed bur is an important exciting source of signals. For the authors, the combination of the vibration waves and the sound pressure data is closely related to the bone density, and that provides information to the surgeon about the relative position between the bur blade and bone. The same authors demonstrated that with the use of features derived on these vibration and sound signals, it is possible to identify automatically different bone penetration states during the operation. Secondly, Suhn et al. [30] investigated changes of surface textures during a robotic palpation task in order to differentiate synthetic materials. Based on the use of the acquired vibro-acoustic (VA) signals originating from a palpation interaction, the authors analyzed the influence of two contact parameters during palpation: angle and velocity. The utilisation of VA signals is a new method of fusion medical information (non-visual) that may help surgeons differentiate biological tissues when indirectly the tissue is palpated using the surgical instrument. Suhn et al. describe that VA waves are obtained through a sensor located at the distal end of the surgical instrument. Therefore, with this described technique, the VA sensor has the capability to collect palpation data of the tissues from the proximal end of the surgical instrument, thereby eliminating the requirement for direct interaction between the sensor and the tissues. The base of the feature extraction methodology followed by [30] included a feature selection routine (based on simple band-pass filters) to attempt to sort out the best and worst features; the utilisation of the discrete wavelet transform to compute stationary spectrum; and the use of histogram techniques to obtain several indicators derived from the instantaneous dominant frequencies, spectral energy and statistical values.

In this article, as an extension of the work presented by [30], we propose a perspective of feature engineering that is based solely on the VA information, in the spectral analysis derived of the Fast Fourier Transform (FFT) algorithm, and no feature selection routine is required. Additionally, we introduce the utilisation of unsupervised data analysis to visually explore our features, find clusters and identify patterns among the features, and describe potential latent structures among the vibro-acoustic data. The unsupervised data analysis is employed to assess the capability of the feature engineering approaches in achieving high performance in a classification task of the synthetic materials evaluated. The proposed methodology helps to overcome the limitations in [30] that could arise when attempting to analyze a greater variability of materials (specially, biological tissues), as there would be no need to perform a feature selection routine for each of them. The findings of this research also hold significant potential for the analysis of larger volumes of medical data. We expected to contribute new insights to the field of vibro-acoustic information analysis in the context of minimally invasive surgery and aid in the task of identifying biological tissue by using features-based algorithms derived from the interaction between the surgical instrument and the material.

## 2. Materials and Methods

To discern the information capable of distinguishing materials, we need to establish a systematic approach. Our approach diverges from [30] in the data transformations applied; it offers distinct perspectives on the nature of the data as well as on dimensionality reduction and clustering techniques.

In this section, we outline the data to be used, delve into the feature extraction procedures, introduce the dimensionality reduction techniques, and specify the metrics employed to assess the effectiveness of the clustering achieved by these methods.

**Figure 1:**
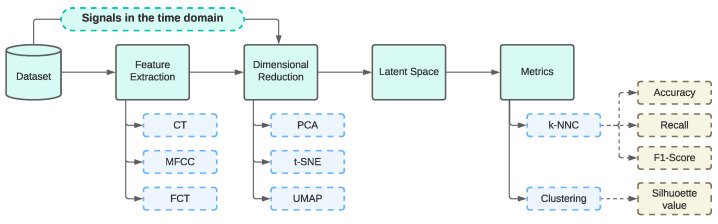
Workflow diagram representing the different stages (feature extraction, dimensional reduction, and classification and clustering metric acquisition).

In Figure 1, the workflow used in this research is illustrated. This process encompasses the treatment of the analysed signals, including a study in the time domain, concurrently with feature extraction. Subsequently, dimensionality reduction techniques are applied, and finally, validation metrics are obtained.

In the final stage, all the sections presented in Figure 1 will allow for comparing the performance achieved and evaluating the potential of these feature extraction techniques in combination with unsupervised dimensionality reduction models for classifying VA signals. Additionally, incorporating a temporal domain analysis is considered to determine whether applying these feature extraction techniques as an intermediate step between the signals and the algorithms is necessary.

### 2.1. Dataset

The data utilised in this study are sourced from the research presented in [30]. These are 900 VA signals captured from the interaction between a surgical instrument and synthetic materials. These signals were collected using the Franka Emika Panda robot with an acoustic–vibration sensor positioned at the end of the robot’s arm. The sensor recorded the signals at a sampling rate of 44.100 samples per seconds that allows exploring up to 22.050 Hz of the VA interaction.

The synthetic materials the robot interacted with included Carpet, a Dots-Engraved Scrubber, and a Smooth Sponge. These materials were engaged by the robot’s arm using various setups particularly with different angles and speeds. The setups used for the robot’s interactions with the synthetic materials were the following:

- Angle of $30^{\circ }$ with a speed of 33 mm/s (100 repetitions for each material);
- Angle of $30^{\circ }$ with a speed of 66 mm/s (100 repetitions for each material);
- Angle of $70^{\circ }$ with a speed of 66 mm/s (100 repetitions for each material).

### 2.2. Feature Extraction

Analysing the VA signals for the feature extraction is a crucial step. Even though the VA signal is unique, the most important information is contained in the signal and must be extracted. The utilisation of several transformations is described. As can be seen in Figure 1, the feature extraction may consist of three different techniques. Each one represents a way for representing numerically the data in order to understand and manipulate them.

Firstly, the traditional technique of Cepstrum Transform (CT) is a mathematical tool that is well known in signal processing and analysis. The idea behind CT is to enable the separation of exciting sources contained in a VA signal and also distinguish harmonic components and resonant frequencies that might overlap in the spectral domain [31]. The feature extracted using CT intend to capture the exciting sources and how the sound is modified by the medium through which it travels, such as a speech signal passing through the unique anatomy of the individual’s vocal tract. In the context of geophysical data, the CT has been used primarily for the characterisation of earthquake sources and explosion signal analysis [32]. In medical data, CT has been utilized for processing and analysis of the speech of patients with vocal fold scar and sulcus vocalis searching dysphonia patterns [33].

Secondly, the traditional bio-inspired algorithm known as Mel Frequency Cepstral Coefficients (MFCC) is commonly used in the context of speech recognition and speaker verification especially under noisy conditions since they are noise robust and invariant both to additive and convolutional noise [34,35,36]. The idea of MFCC is to capture the most salient features based on a nonlinear frequency scale derived from the human peripheral auditory system, which would reflect how our hearing perceives the VA signal in terms of spectral power density in Mel frequency bands. In the context of medical data, MFCC has been used for extracting relevant features in cardiovascular disorder tasks for early detection [37].

Thirdly, the Fast Chirplet Transform (FCT) algorithm is useful for analysing VA signals due to their abrupt frequency changes over short time intervals. The idea is to capture the inherent variability in such signals [38]. The FCT is based on signal expansions over the modulated transient events in amplitude and frequency, which is also known as chirplets. The utilisation of the FCT in signal processing includes object detection in radar and sonar images [39], characterisation of wireless communication signals [40], seismic signal analysis [41], and in the context of medical data, using electrocardiogram signals analysis for the automatic detection of cardiac arrhythmias [42].

### 2.3. Feature Clustering and Visualisation

Due to the multidimensional nature of the features obtained through these methods mentioned above, different dimensionality-reduction algorithms (DRAs) were applied to cluster, visualise, and identify certain clustering patterns that hint at the effectiveness of these transformations in characterising the evaluated synthetic materials. As output of the DRAs, a latent space is generated to represent in 2D or 3D dimensions the similarities between the data.

Three different DRAs were tested. The first one, Principal Component Analysis (PCA) [43], was used as a baseline. This linear algorithm was applied to transform the extracted features, whether from CT, MFCC, or FCT, into principal components, aiming to maintain the signal’s primary variance in a reduced-dimensional space. PCA has been widely used in various fields. In the field of genomics, it has been applied in studies of natural selection to identify genome regions that have undergone local adaptation without the need to define populations beforehand. Correlations between genetic variants and principal components obtained through PCA analysis are utilised in [44]. In the area of structural biology, PCA is employed to analyse datasets of disordered proteins and achieve a more condensed representation of their structural and functional properties [45].

In contrast, the other two methods used, t-Distributed Stochastic Neighbour Embedding (t-SNE) [46] and Uniform Manifold Approximation Projection (UMAP) [47], are not only nonlinear algorithms but they also consider all the features extracted through the feature extraction methods. t-SNE does not assume a linear relationship between input and output variables. For this reason, t-SNE is especially useful when researchers want to visualise complex patterns among features in a dataset. The algorithm reduces the dimensionality of the data while preserving similarity relationships between instances [46]. t-SNE has found applications in a wide range of fields. In the context of medical data, an approach based on the rapid visualisation of large volumes of data from brain magnetic resonance imaging was proposed in the article [48]. In the field of robotics, particularly in robotic surgery, studies like [49] have presented consistent results.

UMAP has become one of the most widely used nonlinear DRAs for visualising complex patterns among features in a two- or three-dimensional space. Although UMAP shares similarities with t-SNE, it has some advantages over other algorithms. UMAP also preserves similarity relationships between instances, but it employs a graph-based optimisation technique to find a low-dimensional representation that preserves both the local and global structure of the high-dimensional space [47]. This allows UMAP to be particularly effective in identifying more complex patterns in the data, such as nonlinear clusters. UMAP has been proven to be the most effective technique in exploratory visualisations of data, and it has found applications in a variety of fields. In the context of medical data, it serves as a key tool for discovering clinically relevant disease subtypes from gene expression data, as demonstrated in [50].

### 2.4. Validation and Metrics

The validation stage involved the utilisation of two distinct metric approaches. The first is associated with the k-Nearest Neighbour Classification (k-NNC) algorithm [51], where the latent space resulting from PCA, t-SNE, and UMAP was employed as input. In this instance, metrics such as Accuracy, Recall, and F1-score were calculated. Previously, the k-NNC algorithm has been employed in studies related to the detection of cardiac arrhythmias using electroencephalogram signals, as demonstrated in the research by Tuncer et al. (2022) [52]. Similarly, it has been used for the analysis of electrocardiograms to discriminate among signal classes as evidenced in the study conducted by Adam et al. (2018) [53].

Simultaneously, the Silhouette value was applied as a clustering metric with the aim of evaluating data cohesion based on their respective labels [54]. In a medical context, the Silhouette value has been employed as a validation metric in rehabilitation studies involving electromyography [55]. Furthermore, in another study [56], this metric was used to assess the quality of clusters generated through the analysis of single nucleotide polymorphism genotyping.

With the intention of measuring the capability of features extracted from VA signals, our approach initially focuses on their ability to cluster based on similarities and subsequently shifts its focus to identifying separating planes among the classes of studied materials. The selection of metrics such as Accuracy, Recall, and F1-score is justified due to their capacity to intricately assess the model’s performance in terms of precision, the ability to detect positive cases, and the balance between precision and recall. Additionally, we have incorporated the analysis of the Silhouette value to evaluate the cohesion or dispersion of data within the identified groups, providing valuable insights into the quality of clustering and the separation between groups. Overall, the choice of these metrics offers a comprehensive assessment of the capacity of the extracted features to cluster and classify the data, which is crucial for the success of our analytical approach.

### 2.5. Parameter Adjustments

Regarding the feature engineering component above described, based on different methods to extract good features from the input VA signals, one of the key parameters is the number of points of Fast Fourier Transform (FFT) for the CT estimation [31]. Generally, a higher number of FFT points provides higher spectral resolution, meaning that more precise frequencies can be identified. However, processing a larger number of FFT points also requires additional computation times. In this study, an FFT points number of $2^{14}$ was used to calculate the power spectral density of the input VA signal, allowing for the necessary spectral resolution in order to obtain the CT for the presented results.

For the MFCC algorithm, one of the most important parameters to select is the number of filters of the Mel filter bank to use. Mel filters are necessary to decompose the VA signal into unequally spaced frequency bands in the Mel scale, which better reflects how the human auditory system perceives frequencies in a nonlinear way. The number of Mel filters was selected based on conventional practices in signal processing, which commonly utilise between 20 and 40 Mel filters [57]. Following this, the results will be presented using 30 Mel filters, which is a value empirically chosen to strike a balance between data resolution and computational complexity. This number of filters renders a good balance between spectral resolution and the calculation time required for signal processing.

The FCT is defined through several hyperparameters that must be adjusted for optimal performance [38]. The selection of these parameters was based on a combination of literature recommendations, preliminary testing, and the specific characteristics of the VA signals under study—specifically the following:

- The duration of the longest Chirplet was set to 1 time unit;
- The number of octaves for the transform was fixed at 10;
- The number of chirps per octave was set to 12;
- The final smoothing was set to 0.001.

In the process of training and testing the k-NNC, first, the original dataset was divided into two parts: 70% for training and 30% for testing. Next, the training stage of the k-NNC model was conducted, where the number of neighbours equal to 2 was defined.

## 3. Results

In this section, the results are presented in two ways—first, the visual performance of latent spaces, and lastly, the metrics that show the clustering.

**Figure 2:**
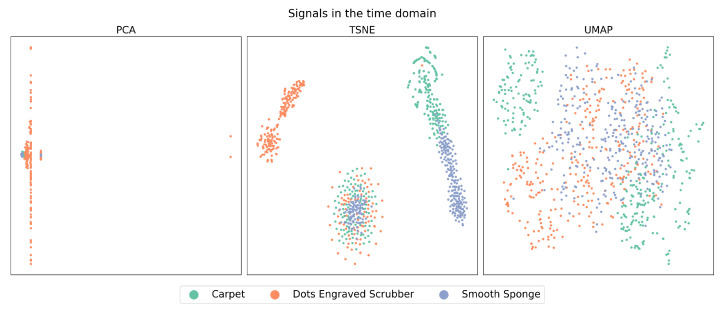
Latent spaces of signals in the time domain. Theses spaces are coming from PCA, t-SNE and UMAP algorithms.

**Figure 3:**
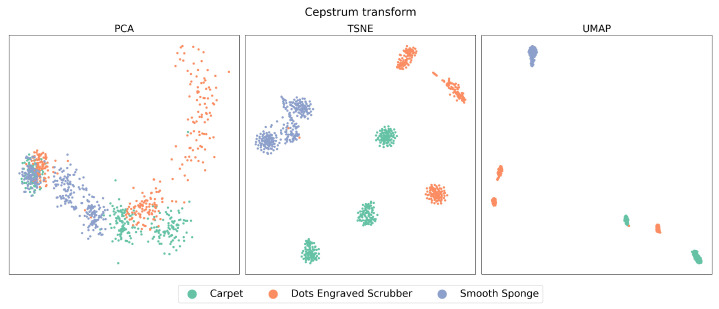
Latent spaces of CT signals. Theses spaces are coming from PCA, t-SNE and UMAP algorithms.

Figure 2 and Figure 3, which we will refer to as the “latent spaces Figures”, show the entire dataset in the latent spaces produced by DRAs in the time domain and CT, respectively. In these spaces, in the case of PCA, each point represents the two principal components or features that contain the most representative information from the data. In contrast, t-SNE and UMAP represent all approximate features and project them into a 2D space.

**Figure 4:**
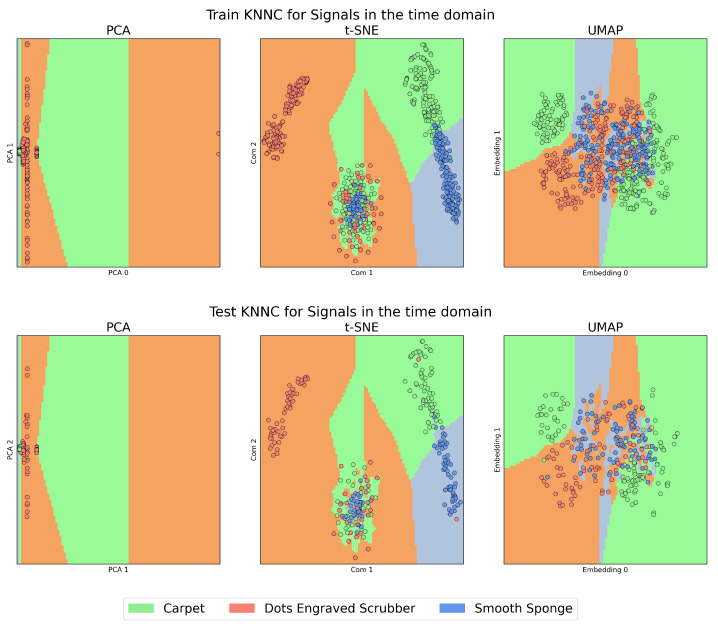
Clustering performance with k-NNC. Comparison of training and testing using k-NNC on PCA, t-SNE, and UMAP for signals in the time domain.

**Figure 5:**
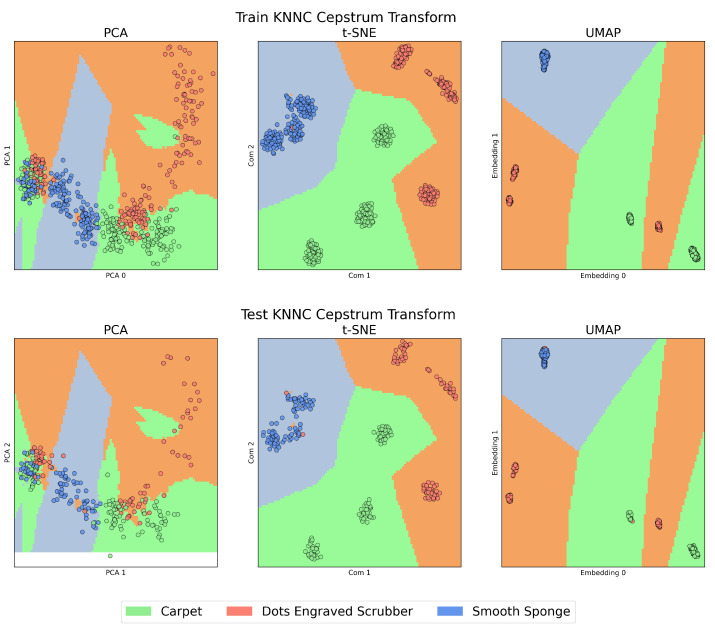
Clustering performance with k-NNC. Comparison of training and testing using k-NNC on PCA, t-SNE, and UMAP for CT signals.

On the other hand, Figure 4 and Figure 5, which we will call the “KNNC Figures”, present the training (above) and testing (below) stages of the KNNC algorithm for signals in the time domain and those transformed by CT. In all these figures, the colour green represents the Carpet material, orange is used for the Dots-Engraved Scrubber, and blue indicates the Smooth Sponge. Specifically, in the KNNC figures, each coloured region designates the expected location for the signal representation of each material.

In Figure 4, a high presence of overlap is observed, which means that the method failed to find a separation plane that discriminated between the characteristics of each materials.

The case of t-SNE, to some degree, showed a clustering tendency, especially in the upper-left regions that correspond to features belonging to Dots-Engraved Scrubber tissue, and in the right region of the plot for the Smooth Sponge and Carpet tissues. However, in the central lower zone of the t-SNE plot, the data exhibited a high level of overlap and dispersion among different classes, which could indicate poor clustering performance. These results were even more compromised when the outcomes obtained with PCA were examined, where it was challenging to identify clear groups for each label.

When examining the features extracted through CT, as can be seen in Figure 5, a notable improvement can be observed in each of the studied algorithms compared to the previous case, displaying a considerably higher level of data clustering. When comparing PCA, t-SNE, and UMAP, it is evident that PCA exhibits the lowest performance in relation to the three aspects mentioned above. High overlap is observed, especially when reviewing the left zone of the plot, and the highest data dispersion, as seen in the case of the Dots-Engraved Scrubber tissue. The main differences between t-SNE and UMAP are related to UMAP’s superior ability to cluster data belonging to the same label with lower dispersion. More defined and compact areas are observed in the UMAP plot, whereas t-SNE achieves clear data clouds for each label but with considerably higher dispersion.

Unlike signal analysis in the time domain, for the cases of t-SNE and UMAP, CT is capable of generating separation regions between classes both in training and in tests. An important characteristic when comparing UMAP with t-SNE (cepstrum) is the result of the dispersion between the groups of clusters that are formed, which is more evident in the case of tests with UMAP. Additionally, anomalous data appear in the case of the test with the PCA method, which is outside any region defined by the algorithm in its training stage. This could be due to the presence of nonlinearities in the data from the latent space generated by PCA.

Regarding the results presented below, the initially defined parameters for each feature extraction technique, DRA, and classification method will be mentioned in the first instance. For the classification task, the Accuracy, Recall, and F1-score from the confusion matrix are used, and for the clustering metrics, the Silhouette value is employed. These results are separated by the feature extraction method. Table 1 shows the metrics obtained for the case of only VA signals in the time domain. Then, Table 2 presents metrics obtained for CT. Table 3 shows metrics obtained for MFCC. Table 4 and Table 5 present metrics obtained for the case of the feature ensemble.

In the case of the VA signal in the temporal domain, the metrics summarized in Table 1 indicate that they do not achieve values over 85%. At first glance, this might seem like a performance considerably high, but by analyzing the Silhouette values estimated, it revealed that the values for all three DRAs are very close to zero. This indicates uncertainty in data clustering and the presence of ambiguity in training.

In the case of CT, considerably higher percentages are observed compared to the previous case, surpassing 99%, as shown in Table 2. However, a difference is observed when analyzing the clustering metric. UMAP yields the highest clustering metric value of approximately 0.61 among all the combinations of feature extraction techniques and DRAs considered in this research. In contrast, for the CT with t-SNE combination, while excelling in classification, it demonstrates a clustering metric value of around 0.49. By analyzing the case CT-PCA, the results in terms of classification and clustering metrics were inferior to those obtained for VA signals in the temporal domain.

The results obtained from MFCC coefficients show classification metrics of around 90% for PCA, around 98% for t-SNE, and around 99% for UMAP, as can be seen in the metrics in Table 3. Regarding the clustering metric, it can be observed that despite obtaining a percentage considerably superior, the Silhouette value demonstrates that PCA lacks the necessary certainty to correctly define points associated with their respective labels. In the cases of t-SNE and UMAP, the values are higher than those of PCA, but they cannot be considered high, since they are closer to 0 than to 1. This is primarily due to the greater overlap compared to the CT case.

When we analyze the metrics obtained from FCT shown in Table 4, Accuracy, Recall, and F1-score values above 90% are observed in some cases for both PCA and t-SNE. However, in the case of UMAP, the results are even lower than those obtained in the analysis of signals in the temporal domain. The clustering metric confirms the aforementioned, as the values are close to 0, reflecting the overlap mentioned during the subjective analysis phase.

Concerning the feature ensemble technique, the results reflect the contribution already identified in the initial analysis, highlighting the use of features provided by the MFCC coefficients. This is evident when comparing the results of PCA in its classification metrics as seen in Table 5, where values close to 90% are obtained. However, the values for t-SNE and UMAP do not surpass those obtained with CT and even decrease, especially in the case of UMAP. Regarding the Silhouette value, it is possible to find the second-best result, which corresponds to 0.56 and belongs to UMAP.

## 4. Discussion

### 4.1. Dimensional Reduction Analysis

When analyzing the latent space of signals in the time domain (see Figure 2), the data have limited ability to cluster according to their respective labels. In the case of t-SNE, it can be seen that the “Dots-Engraved Scrubber” material manages to cluster some of its signals, but not all, as there is a cluster of points associated with the three materials in the lower centre. This was expected due to the high variability of features derived from amplitude changes over time, which are used as input in the dimensionality reduction algorithms (DRAs).

As can be observed in Figure 3, the change is remarkable, especially in the cases of t-SNE and UMAP, which clearly managed to create well-defined data clusters for each material. Furthermore, it is evident that UMAP, in addition to grouping the data, exhibits a relatively low level of dispersion between clusters compared to t-SNE. This aspect will be relevant in a subsequent analysis to determine which method allows for a more effective classification of these materials.

In both cases, PCA failed to efficiently cluster the data according to the corresponding material. This could indicate the nonlinear naturc present in these signals.

### 4.2. Validation

The results obtained when performing the classification process using KNNC support the preliminary analysis conducted in the dimensionality reduction stage, as high-performance metrics are achieved for the case of CT compared to those obtained for the time domain VA signals. This result underscores the importance of the extracted features, which is similarly reflected in the MFCC metrics.

It is interesting to note that CT demonstrates one of the most outstanding performances. This remarkable performance can be directly related to the predominant applications of CT in signals such as speech and the study of seismic signals, as mentioned in previous sections. CT has shown good results in capturing how VA signals are modified depending on the medium through which they are transmitted; in this case, the surgical instrument acts as the signal transmission medium.

All of this suggests that for future experiments, it is crucial to take into consideration the type of instrument that will be used. It should be noted that if the same experiment is conducted with a different surgical instrument as the medium for propagating VA signals, the results may vary.

Although the metrics obtained from KNNC demonstrate high performance in various combinations of feature extraction techniques and DRAs, it is the inclusion of the Silhouette value clustering metric that best reflects the efficiency of CT-UMAP as a combination for this methodology compared to other combinations. This is because KNNC provides the regions for each material within the latent spaces but does not offer information about the dispersion of data within those regions. Thus, incorporating the Silhouette value complements this information by allowing us to assess the cohesion and separation of clusters, which is essential for a more comprehensive understanding of data distribution in the latent space.

The main reason why this metric is not even higher is because for both the “Dots-Engraved Scrubber” and “Smooth Sponge” materials, multiple clusters of points were generated. This suggests that certain materials are more susceptible to how data are captured, which may be due to the angle or speed. This idea is further supported by the analysis conducted in [30], which establishes that the way signals are acquired influences the type of features obtained.

Just as the metrics obtained for CT-UMAP or MFCC-UMAP exhibit the highest performances, the case of FCT is entirely different, showing a performance very similar to that of time domain signals. This may indicate the absence of frequency modulations in the analyzed signals. FCT, relying heavily on variations in frequency over time, is less effective when these modulations are scarce or practically non-existent.

### 4.3. Methodology

The methodology employed has demonstrated high performance with certain combinations of feature extraction and DRAs, which fulfills the main objective of this research. In the previous study reported in [30], the methodology and analysis were focused on the selection of specific features for each material. This involved preprocessing and a feature selection routine based on simple filtering of the VA signals as well as processing even more complexity based on the utilisation of the Continuous Wavelet Transform (CWT). This approach not only requires more computational analysis time but also becomes limited when attempting to incorporate new types of materials.

This last point highlights the valuable contribution of unsupervised data analysis to visually explore our features, since it paves the way for training deep neural networks and other machine learning models in the future. This perspective is grounded in the fact that by avoiding the use of specific feature selection techniques, the need to tailor and adapt the methodology to each dataset or type of material (synthetic or biological tissue) is eliminated, resulting in significant time and resource savings. Avoiding the application of these specific techniques not only simplifies the current analysis process but also vastly expands the potential of the proposed methodology to facilitate the training of deep neural networks and other automated approaches in the future.

The current methodology can be generalised in a variety of applications in robot-assisted surgical interventions. Through the efficient classification of materials based on VA signals, surgeons can gain valuable information about the tissues they are working with. This can improve the accuracy of tissue characterisation and ultimately lead to better surgical outcomes by providing surgeons with an additional tool to make informed decisions during procedures. The methodology also stands out for its ability to adapt to different surgical instruments, making it versatile and applicable in various surgical settings.

While the methodology proves to be promising in material classification based on VA signals, its implementation in real surgical environments poses regulatory, technical, and operational challenges that must be addressed before widespread adoption. Compliance with regulations, effective integration, and adaptation to procedural variability are critical considerations for the success of this technology in the field of minimally invasive surgery.

The deployment of a system based on the current methodology requires a VA sensor that complies with all the required regulations. Overcoming this challenge will enable continuous surgery data collection, ongoing model improvement and, therefore, good features that are more robust and invariant.

## 5. Conclusions

In summary, the main objective of this research is to develop a model capable of clustering and classifying synthetic materials without relying on signal preprocessing stages, or feature selection routines based on filtering, unlike previous research in [30]. We aim to analyse the potential of combining feature engineering with valuable methods of unsupervised dimensionality reduction to classify VA signals generated by direct contact between surgical instruments and synthetic materials. Furthermore, the utilisation of a VA sensor has the capability to collect the palpation data of the materials from the proximal end of the surgical instrument, eliminating the requirement for direct interaction between a sensor and tissues.

The results demonstrate that the combination of feature extraction using CT and dimensionality reduction with UMAP provides the best performance compared to other evaluated techniques. We achieved a performance close to 99% in classification metrics (Accuracy, Sensitivity, and F1-score) and a Silhouette index value of 0.61. The CT-UMAP combination outperforms all others.

Although the results may be promising, there is room for improvement, especially regarding the Silhouette index. This may be due to the formation of multiple clusters for the same material, which is a limitation that can be addressed in future work.

In future studies, it would be interesting to test our methodology with a variety of state-of-the-art techniques, as employed in [30], as well as with the use of the Continuous Wavelet Transform. Furthermore, it would be valuable to explore other innovative methods, such as the Autoregressive Models and Variational Autoencoders. Comparisons and additional exploratory visualisations would provide a deeper understanding of the effectiveness and potential of our methodology. They will also help determine if our approach is the most suitable for addressing specific biological tissues or if there are alternative approaches that could yield even more promising results based on the fusion of medical information extracted from VA waves.

These findings suggest that the feature engineering based on VA signals could be utilised in the automatic classification of biological tissues in the context of minimally invasive robotic surgery. For this purpose, we expect to conduct new research using biological tissues with two potential approaches. The first approach is associated with the possibility of expanding the number of examples and being able to distinguish between different tissues, such as bone, cartilage, tendons, etc. The second approach is related to being able to detect differences within the same tissue with the idea of identifying tumours, which holds significant potential in defining more precise resection areas, ultimately having a substantial impact on patients’ lives.

## Figures and Tables

**Table 1:** Clustering performance for signals in the time domain. PCA is compared with t-SNE and UMAP. Accuracy, Recall, and F1-score are reported as classification metrics, and the Silhouette value is reported as a clustering metric.

Dimensional Reduction Algorithm	Metrics			
	**Accuracy**	**Recall**	**F1-Score**	**Silhouette Value**
PCA	0.849	0.833	0.834	0.020
t-SNE	0.826	0.811	0.811	0.248
UMAP	0.744	0.726	0.704	−0.031

**Table 2:** Clustering performance for CT. PCA is compared with t-SNE and UMAP. Accuracy, Recall, and F1-score are reported as classification metrics and the Silhouette value is reported as a clustering metric.

Dimensional Reduction Algorithm	Metrics			
	**Accuracy**	**Recall**	**F1-Score**	**Silhouette Value**
PCA	0.741	0.719	0.721	0.072
t-SNE	0.993	0.993	0.993	0.488
UMAP	0.993	0.993	0.993	0.606

**Table 3:** Clustering performance for MFCC. PCA is compared with t-SNE and UMAP. Accuracy, Recall, and F1-Score are reported as classification metrics, and the Silhouette value is reported as a clustering metric.

Dimensional Reduction Algorithm	Metrics			
	**Accuracy**	**Recall**	**F1-Score**	**Silhouette Value**
PCA	0.909	0.904	0.903	0.004
t-SNE	0.985	0.985	0.986	0.362
UMAP	0.993	0.993	0.993	0.220

**Table 4:** Clustering performance for FCT. PCA is compared with t-SNE and UMAP. Accuracy, Recall, and F1-Ssore are reported as classification metrics and the Silhouette value is reported as a clustering metric.

Dimensional Reduction Algorithm	Metrics			
	**Accuracy**	**Recall**	**F1-Score**	**Silhouette Value**
PCA	0.907	0.896	0.897	0.163
t-SNE	0.923	0.921	0.922	0.106
UMAP	0.716	0.722	0.707	0.013

**Table 5:** Clustering performance for feature ensemble between CT and MFCC. PCA is compared with t-SNE and UMAP. Accuracy, Recall, and F1-Score are reported as classification metrics, and the Silhouette value is reported as a clustering metric.

Dimensional Reduction Algorithm	Metrics			
	**Accuracy**	**Recall**	**F1-Score**	**Silhouette Value**
PCA	0.916	0.911	0.910	0.032
t-SNE	0.993	0.993	0.993	0.481
UMAP	0.989	0.989	0.989	0.560

## Data Availability

Restrictions apply to the availability of these data. Data were obtained from SURAG Medical GmbH and are available from the authors with the permission of SURAG Medical GmbH.

## Abbreviations

The following abbreviations are used in this manuscript:

MIRS	Minimally Invasive Robotic Surgery
VA	Vibro-Acoustic
CWT	Continuous Wavelet Transform
SVM	Support Vector Machines
CT	Cepstrum Transform
MFCC	Mel Frequency Cepstral Coefficients
FCT	Fast Chirplet Transform
DRA	Dimensionality Reduction Algorithm
PCA	Principal Component Analysis
t-SNE	t-Distributed Stochastic Neighbour Embedding
UMAP	Uniform Manifold Approximation Projection
k-NNC	k-Nearest Neighbour Classification
FFT	Fast Fourier Transform
